# Experimental Determination of Solid–Liquid Equilibrium of Calcium in Portland Cement Under Seawater Exposure

**DOI:** 10.3390/ma19102117

**Published:** 2026-05-18

**Authors:** Yong Wang, Jiali Lin, Fuqiang He, Wei Sun

**Affiliations:** School of Civil Engineering and Architecture, Xiamen University of Technology, Xiamen 361024, China; 2322201019@s.xmut.edu.cn (J.L.); 2011110901@xmut.edu.cn (F.H.); 2322201028@s.xmut.edu.cn (W.S.)

**Keywords:** calcium leaching, solid–liquid equilibrium, seawater exposure, Portland cement

## Abstract

Calcium leaching is a key degradation mechanism governing the long-term durability of cement-based materials, particularly in marine environments where multi-ionic interactions significantly alter dissolution behavior. In this study, the solid–liquid equilibrium of calcium in hardened Portland cement exposed to NaCl solution and artificial seawater was experimentally established. A wide range of equilibrium states was achieved by varying the liquid-to-solid ratio, and the corresponding aqueous chemistry and phase assemblage were characterized using ICP-AES, XRD, and SEM-EDS. The results show that both NaCl solution and seawater substantially increase the equilibrium calcium concentration compared to deionized water, with a much stronger effect observed in seawater. In NaCl solution, the equilibrium relationship follows a classical three-stage decalcification process. In contrast, seawater induces coupled dissolution–precipitation reactions due to the presence of Mg^2+^ and SO_4_^2−^, leading to rapid and extensive decalcification within a narrow calcium concentration range. The findings provide important experimental evidence for improving thermodynamic models and predicting the long-term degradation of Portland cement concrete in marine environments.

## 1. Introduction

**Figure 1:**
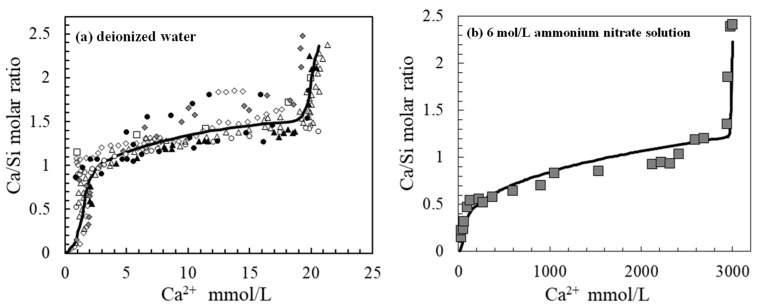
The thermodynamic equilibrium relationship between the calcium ions in solution ((**a**) deionized water [7], (**b**) 6 mol/L ammonium nitrate solution [13]) and the average Ca/Si molar ratio of solid products in hardened Portland cement paste. (For (**a**), different symbols denote experimental data from various literature sources, and the solid line represents the fitted curve.).

Calcium leaching is one of the primary degradation mechanisms affecting cement-based structures, such as hydraulic infrastructures and radioactive waste repositories, where long-term durability is essential [1,2,3]. It is generally considered a diffusion-controlled process, in which calcium ions gradually migrate from the solid matrix into the surrounding solution [4]. Due to the inherently slow kinetics of diffusion, experimental investigation of calcium leaching is often time-consuming [5]. Therefore, predictive numerical models have been widely developed to assess long-term performance [5,6,7,8]. In these models, the thermodynamic equilibrium between pore solution and solid hydrates plays a fundamental role, as it governs the calcium concentration gradient that drives ionic diffusion. Gérard et al. [4,7] experimentally established this equilibrium relationship, correlating the calcium concentration in solution with the Ca/Si molar ratio of the solid phases, as illustrated in Figure 1a.

The equilibrium curve shown in Figure 1a reflects the sequential dissolution of calcium-bearing phases during leaching. Portlandite dissolves first and is completely depleted at a calcium concentration of approximately 20 mmol/L. This is followed by the decomposition of AFm phases and progressive decalcification of C-S-H as calcium concentration decreases from 20 to 1.5 mmol/L. Ultimately, at extremely low calcium concentrations, C-S-H transforms into silica gel [9,10,11,12]. Since the concentration gradient is the primary driving force, accelerated leaching techniques are often employed to shorten experimental duration. For instance, the use of 6 mol/L ammonium nitrate solution significantly increases the equilibrium calcium concentration to approximately 2320 mmol/L (Figure 1b), thereby greatly enhancing calcium diffusion rates [9,13].

Previous studies [14,15,16] on calcium leaching have mainly focused on soft-water conditions. In contrast, research on marine-exposed concrete has predominantly emphasized chloride ingress due to its role in steel corrosion [17,18,19]. However, calcium leaching remains a fundamental degradation mechanism in both environments and should not be neglected [20]. Experimental observations have demonstrated a strong correlation between calcium leaching and chloride penetration, with similar penetration depths reported in marine-exposed concrete [21,22,23,24]. The presence of chloride ions can significantly modify the solubility of calcium-bearing phases, indicating that calcium leaching behavior in marine environments differs substantially from that in pure water systems [25,26,27].

Numerous studies have confirmed that saline solutions enhance the decalcification of cement hydrates [3,28,29]. The solubility of portlandite increases with chloride concentration, reaching a maximum value approximately 138% higher than that in deionized water at a chloride concentration of 0.5 mol/L [28]. This enhancement has been attributed to the formation of weak calcium–chloride complexes [29]. In deionized water, dissolution of C-S-H gel occurs incongruently, and the equilibrium calcium concentration depends on its Ca/Si ratio [30,31]. This behavior persists in NaCl solutions, although calcium solubility is elevated [28,29,32]. Sugiyama [32] observed that sodium sorbed onto the C-S-H phases and some sodium replaced calcium in C-S-H so that the release of calcium was enhanced. Hill et al. [33] compared the effect of salt cation type (Li, Na, and K) on the dissolution of C-S-H; anomalously high calcium concentrations were observed in the presence of LiCl. Such replacement is ruled by the effective cation exchangeable sites in C-S-H gel, where sodium is present exclusively and in higher quantities at low C/S ratios than at high ones [34,35]. Experiments also show that the effect of NaCl concentration on the enhancement of calcium release from C-S-H gels is stronger at a lower C/S ratio [32], which even gives higher Ca concentrations at high NaCl levels (>0.3 mol/L) than the one with a higher C/S [33]. Tao et al. [3] explained it from the perspective of dissolution free energy: the thermodynamic characteristics of Ca dissolution in seawater are similar to those in pure water but with lower free energy, and C-S-H with higher C/S features lower dissolution free energy.

In addition to Na^+^ and Cl^−^, seawater contains other ions such as Mg^2+^ and SO_4_^2−^, which can further alter the thermodynamic behavior of calcium dissolution [25,26]. Magnesium, especially in combination with sulfate, has a particularly detrimental effect on cement stability [28,36]. Although the penetration depth of Mg^2+^ and SO_4_^2−^ is generally limited, these ions can rapidly react with cement hydrates, leading to the formation of brucite and magnesium silicate hydrate (M-S-H), accompanied by extensive calcium release [20,37,38].

Despite extensive studies on C-S-H dissolution in saline environments, a comprehensive solid–liquid equilibrium relationship for Portland cement exposed to seawater is still lacking. Existing models typically rely on equilibrium data obtained in pure water (Figure 1a), which cannot adequately describe the complex behavior in marine conditions [39]. Therefore, this study aims to experimentally establish the solid–liquid equilibrium relationship of Portland cement in artificial seawater. Considering that chloride ions dominate in the interior regions of marine concrete, NaCl solution is also investigated for comparison. Furthermore, the phase evolution during the decalcification process is systematically characterized. This study will systematically compare the thermodynamic equilibrium behavior of ordinary Portland cement paste in sodium chloride solutions and artificial seawater under controlled conditions. The aim is to elucidate the unique mechanisms underlying calcium leaching and phase evolution in high-salinity marine environments, thereby providing crucial experimental evidence for the development of models to predict the long-term deterioration of concrete in marine infrastructure.

## 2. Experimental

### 2.1. Materials and Sample Preparations

Portland cement (P.I 42.5) supplied by Fushun Cement Co., Ltd. (Fushun, China) was used in this study. The cement was produced by grinding clinker with gypsum, and its chemical composition is listed in Table 1. Cement paste with a water-to-cement mass ratio of 0.5 was cast in sealed plastic bottles. After demolding (after 24 h from casting), the specimens were cured in saturated calcium hydroxide solution at (20 ± 1) °C for 180 days to ensure sufficient hydration. The use of saturated calcium hydroxide solution ensures a stable high-pH environment throughout curing, eliminating the risk of calcium leaching or carbonation and providing a well-hydrated cement paste for subsequent experiments.

The well-cured cement paste (WCP) was then ground and sieved to obtain powder with a particle size smaller than 75 μm (200 mesh) for subsequent exposure tests. To prevent carbonisation of the specimens during grinding, isopropyl alcohol was added to the mortar throughout the process to carry out wet grinding; the grinding time for each batch was strictly controlled at 20–25 min to ensure uniform fineness across all batches. Upon completion of grinding, a vacuum funnel was used to separate the solids from the liquid, and the resulting cement paste powder was collected and dried at a constant temperature of 40 °C in a vacuum oven for 3 days.

Two types of exposure solutions were prepared using analytical-grade reagents and deionized water: (i) NaCl solution with a concentration of 0.53 mol/L and (ii) artificial seawater [40]. The composition of the artificial seawater is given in Table 2.

### 2.2. Exposure Procedure and Measurements

To obtain a wide range of equilibrium states, the liquid-to-solid ratio was varied by changing the exposure solution volume from 50 mL to 20,000 mL while maintaining a constant solid mass of 5.000 ± 0.005 g (30 groups). Each exposure group was conducted separately in a sealed glass bottle. The suspensions were magnetically stirred for 30 min every 12 h to prevent particle agglomeration. All experiments were performed at (20 ± 0.5) °C for at least 30 days to ensure equilibrium conditions. After equilibration, use a syringe to withdraw the supernatant from the conical flask, seal it, and store it for subsequent liquid-phase analysis. Solid samples were collected by vacuum filtration and briefly rinsed with deionised water for 15 s to remove surface residual ions; they were then rinsed twice with isopropanol to effectively prevent carbonisation of the samples. The treated samples were thoroughly ground to ensure uniform particle size, then dried at a constant temperature of 40 °C under vacuum for 3 days, sealed, and stored for subsequent solid-phase characterization tests. The solid phase is separated from the liquid phase by vacuum filtration using a 0.45 μm nylon filter membrane.

#### 2.2.1. Mineral Phase Assemblage

X-ray diffraction (XRD) analyses were undertaken using a Bruker D2 Phaser (Bruker, Billerica, MA, USA) with a LynxEye detector (Cu Kα, 1.5418). The 2θ range goes from 5 to 65° with a step size of 0.02°, and a time per step of 0.4 s. This leads to a total scanning time of 20 min. The X-ray source was operated at 30 kV and 10 mA. To quantify the absolute phase contents and from there the amorphous content, the internal standard method was applied, and TiO_2_ (Rutile) was used as the internal standard.

Thermogravimetric analysis was carried out using a synchronous thermal analyzer (Labsys Evo) from Setaram (Caluire-et-Cuire, France). Around 25 mg of the ground solid sample was placed in alumina crucibles. The temperature ranged from 25 to 1000 °C with a heating rate of 10 °C/min under N_2_ atmosphere. SEM/EDS analysis characterization was applied using an Apreo S (Thermo Scientific, Waltham, MA, USA) environment scanning electron microscope on the dry WCP powder. The atom count percentages are averaged from 10 scans of different regions (200 × 200 μm for each area).

#### 2.2.2. Aqueous Phase Analysis

After vacuum filtration, 1 mL of the filtrate was sampled using an electronic pipette and immediately acidified with 2.7 mL of concentrated nitric acid (65 wt%) to prevent precipitation. The solution was then diluted to 50 mL using ultrapure water prior to analysis.

The concentrations of Ca^2+^ and K^+^ were determined using inductively coupled plasma–atomic emission spectrometry (ICP-AES, Ultima 2, HORIBA Jobin Yvon, Palaiseau, France). The pH of the solution was measured using a calibrated pH meter (accuracy ± 0.01). All measurements were conducted in duplicate, and the average values were reported.

## 3. Results and Discussion

### 3.1. Ions in Exposure Solution

**Figure 2:**
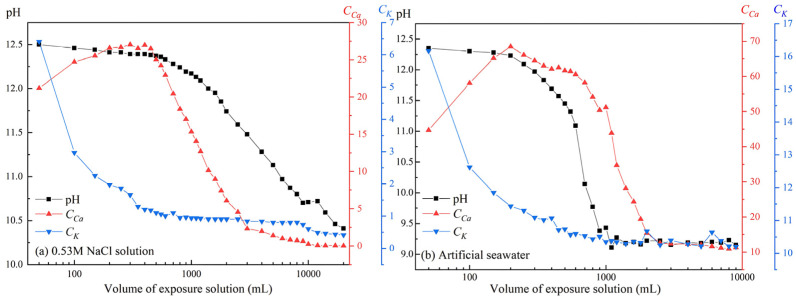
pH and the concentrations of Ca^2+^ (C_Ca_) and K^+^ (C_K_) in two exposure solutions.

Figure 2 presents the variation of the pH and the concentrations of Ca^2+^ (*C_Ca_*) and K^+^ (*C_K_*) in the exposure solutions as a function of solution volume.A strong correlation between pH and *C_Ca_* is observed, indicating that the dissolution equilibrium of WCP is primarily controlled by calcium hydroxide (CH). In both solutions, the initial pH approaches 12.5, corresponding to saturated portlandite conditions. However, the pH in artificial seawater decreases much more rapidly, dropping to approximately 9.2 at 1000 mL, whereas in NaCl solution it remains around 10.4 even at the highest volume (20,000 mL).

Notably, the *C_Ca_* does not decrease monotonically with increasing solution volume. At the early stage, potassium is preferentially released, which temporarily suppresses portlandite dissolution due to ionic strength effects. As *C_K_* decreases, *C_Ca_* subsequently increases until saturation is reached. Compared with deionized water, the maximum *C_Ca_* increases significantly to 27.1 mmol/L in NaCl solution and 68.5 mmol/L in seawater. The increase in NaCl solution is attributed to calcium–chloride complexation, while in artificial seawater, magnesium ions enhance portlandite dissolution by consuming hydroxide ions [22]. This substantial increase in calcium solubility indicates that saline environments can markedly accelerate calcium leaching processes.

### 3.2. Phase Assemblage in WCP

#### 3.2.1. Residual Mass of WCP

**Figure 3:**
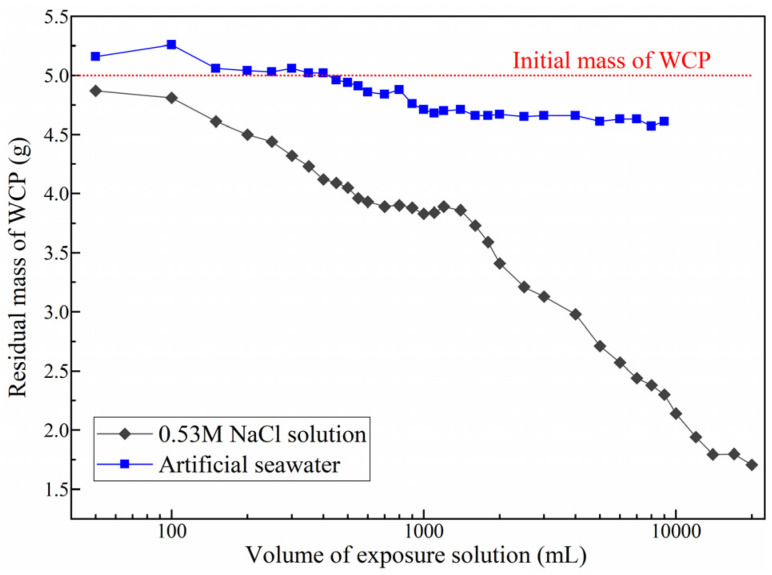
Residual mass of WCP in two exposure solutions.

After reaching equilibrium, the residual WCP was collected, dried to a constant mass, and quantified. The evolution of residual mass in the two exposure solutions is shown in Figure 3. In NaCl solution, the residual mass decreases monotonically with increasing solution volume, indicating continuous dissolution of solid phases. At the highest solution volume (20,000 mL), only 34.2% of the initial mass remains, reflecting significant decalcification and dissolution.

In contrast, under artificial seawater exposure, the residual mass initially increases slightly and even exceeds the original mass before gradually decreasing. At the end of the test, the residual mass remains approximately 2.7 times higher than that in NaCl solution. This distinct behavior suggests that, in seawater, the solid–liquid interaction involves not only dissolution but also substantial precipitation of secondary phases, which partially compensates for mass loss.

#### 3.2.2. Phase Evolution

**Figure 4:**
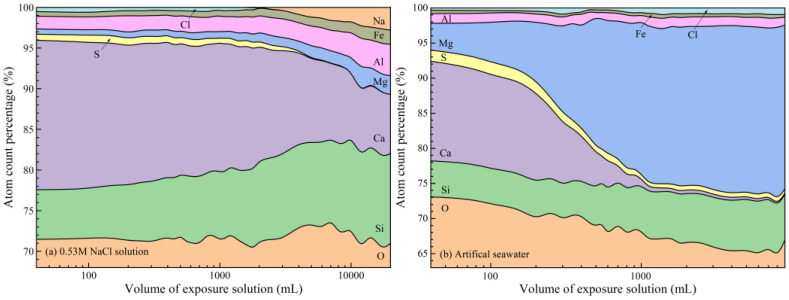
Atom count percentages (EDS analysis) in WCP after reaching equilibrium with different volumes of exposure solutions.

**Figure 5:**
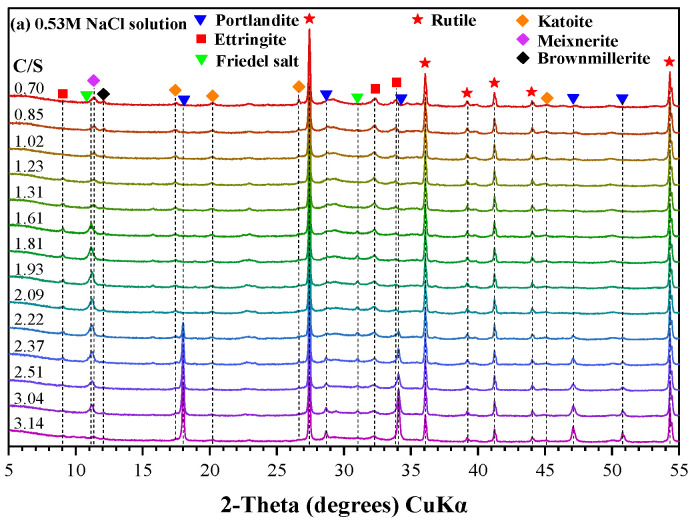

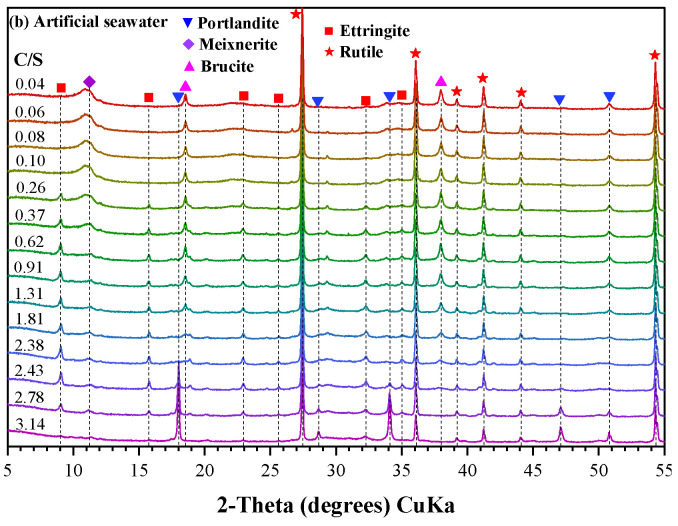
X-ray diffraction patterns of WCP after reaching equilibrium with different volumes of exposure solutions.

Calcium leaching is generally associated with the dissolution of calcium-bearing phases, resulting in mass loss. However, the trends observed in Figure 3 indicate that, particularly in seawater, additional reactions beyond simple dissolution must be considered. The elemental composition (Figure 4, EDS analysis) and XRD patterns (Figure 5, 20% wt. rutile is added as the internal standard for the subsequent Rietveld quantification) provide further insight into phase evolution during exposure.

EDS analysis reveals a significant reduction in calcium content in WCP under both exposure conditions, with a much faster and more extensive loss in seawater. This behavior is primarily attributed to the presence of Mg^2+^ ions. As portlandite dissolves and releases OH^−^, Mg^2+^ reacts with hydroxide ions to form brucite (Mg(OH)_2_). This reaction continuously consumes OH^−^, destabilizing the alkaline environment and thereby promoting further dissolution of calcium-bearing phases. The inverse correlation between brucite formation and portlandite consumption, as observed in XRD patterns, confirms this coupled reaction mechanism. This also explains the relatively lower mass loss but faster pH reduction observed in seawater compared to NaCl solution.

In WCP, chloride ions are considered foreign to the material, which interacts strongly with AFm phases in WCP. In NaCl solution, monosulfoaluminate (AFm-Ms) reacts with chloride to form Friedel’s salt (AFm-Fs), which is clearly detected in both EDS and XRD results. As the solution volume increases and the pH decreases, Fs becomes thermodynamically unstable and gradually decomposes. Its diffraction peaks diminish and eventually disappear when the pH drops to approximately 11.5. A similar trend is observed in seawater; however, chloride signals persist in EDS even after Fs disappears from XRD. This indicates that chloride is no longer present in crystalline form but is instead retained within the solid matrix.

The persistence of chloride in the solid phase under seawater exposure can be attributed to the formation of magnesium silicate hydrate (M-S-H). As Mg^2+^ replaces Ca^2+^ during decalcification, M-S-H gels are formed, which exhibit a strong binding for chloride adsorption. Consequently, chloride ions remain associated with the solid phase even after the dissolution of crystalline chloride-bearing phases. In contrast, in NaCl solution, decalcification of C-S-H leads to the formation of low-calcium silicate gels that preferentially adsorb sodium ions, resulting in an increase in Na signals in EDS analysis.

Sulfur in WCP primarily exists in sulfate-bearing aluminate phases such as AFm-Ms and AFt. In NaCl solution, the formation of Friedel’s salt releases sulfate ions, which subsequently react with aluminates to form secondary AFt in the presence of a high alkalinity and high calcium concentration environment. This is reflected in the increasing intensity of AFt peaks at lower solution volumes. However, as the pH decreases, AFt becomes unstable and eventually dissolves completely when the exposure solution volume reaches 3000 mL, where the pH of the solution is approximately 11.0.

In artificial seawater, the presence of external sulfate ions enhances AFt formation at early stages, leading to an increase in both sulfur content and residual mass. This explains why the mass of WCP can temporarily exceed its initial value. With further exposure and decreasing pH (about 11.0), AFt gradually dissolves. However, due to the high-sulfate environment, minor sulfate-bearing solid solutions persist, resulting in detectable sulfur signals in EDS even at later stages.

### 3.3. Discussion of Solid–Liquid Equilibrium Relationship

**Figure 6:**
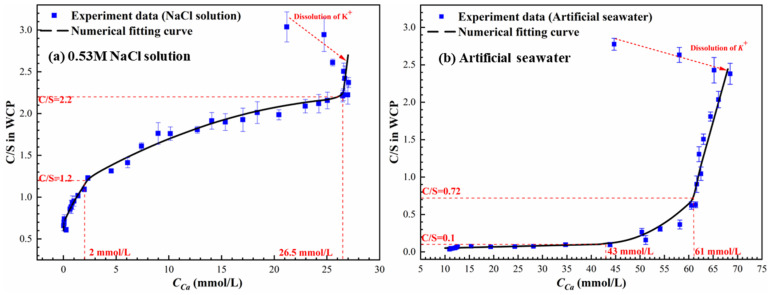
Solid–liquid equilibrium relationship of WCP in two exposure solutions.

Based on Figure 4, the calcium to silicon molar ratios (C/S) obtained from EDS analysis are correlated with the equilibrium *C_Ca_* in solutions (Figure 2), and the resulting solid–liquid equilibrium relationships of WCP in the two exposure solutions are presented in Figure 6.

As shown in Figure 6, an apparent deviation is observed in the high C/S region due to the early alkali-release stage. After the complete depletion of K^+^, the C/S ratio decreases monotonically with decreasing *C_Ca_* in both solutions, although the evolution trends differ significantly. In NaCl solution, the equilibrium is generally consistent with that observed in deionized water (Figure 1a) and can be divided into three stages. In contrast, the equilibrium relationship in artificial seawater deviates markedly from both NaCl solution and deionized water. The C/S ratio decreases sharply within a relatively narrow range of *C_Ca_* and approaches very low values as *C_Ca_* continues to decrease, indicating near-complete decalcification of WCP.

**Figure 7:**
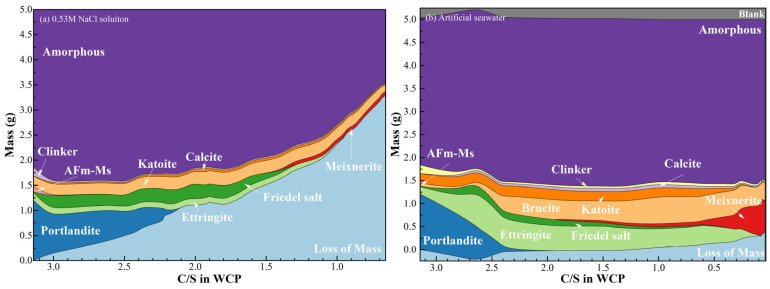
Mineral phase changes in WCP after reaching equilibrium with different volumes of exposure solutions.

To further interpret the equilibrium relationships, Rietveld quantification was applied to the XRD patterns (Figure 5), and the corresponding phase contents were multiplied by the residual mass (Figure 3) to determine the absolute mass evolution of each phase, as shown in Figure 7. As indicated in Figure 7a, upon exposure to NaCl solutions, residual unhydrated clinker in WCP undergoes secondary hydration, resulting in a slight increase in gel-phase content. Meanwhile, AFm-Ms rapidly reacts with chloride ions and is completely transformed into Friedel’s salt. Simultaneously, portlandite dissolution leads to a reduction in the C/S ratio and significant mass loss. However, in artificial seawater (Figure 7b), the presence of Mg^2+^ and SO_4_^2−^ induces additional reactions with hydrated phases, leading to the formation of brucite and AFt. These precipitation processes result in a net increase in solid mass, which can even exceed the initial mass of WCP.

Analysis of Figure 6 and Figure 7 reveals that when the C/S ratio decreases to approximately 2.1, portlandite is completely depleted in both exposure systems. In NaCl solution, the first inflection point in the equilibrium curve (Figure 6a) appears at a C/S ratio of about 2.2, corresponding to a *C_Ca_* of 26.5 mmol/L. This value is significantly higher than that in deionized water, confirming the enhancement of portlandite dissolution by chloride ions. After this point (*C_Ca_* < 26.5 mmol/L), the C/S ratio decreases more gradually as decalcification is mainly governed by C-S-H dissolution. As shown in Figure 7a, when C/S < 2.2, mass loss is primarily associated with the amorphous phase, indicating progressive decalcification of C-S-H. As decalcification proceeds and the pH decreases, Friedel’s salt becomes unstable and starts to decompose when C/S approaches 2.0, further accelerating the reduction in C/S. It is noted that AFt remains relatively stable in the presence of Friedel’s salt. However, once Friedel’s salt is completely decomposed (Ca/Si ≈ 1.4), AFt begins to dissolve and becomes the dominant phase undergoing decalcification, eventually disappearing when C/S reaches approximately 1.0. Consistent with these observations, EDS results in Figure 4a show that chloride and sulfur signals disappear sequentially at C/S values of 1.4 and 0.95, respectively. This corresponds well with the decomposition of Friedel’s salt and AFt. The dissolution of these phases results in significant calcium loss and the removal of chloride and sulfur, while the relative aluminum content increases, indicating incongruent dissolution behavior.

In artificial seawater, the equilibrium relationship (Figure 6b) exhibits a markedly different evolution pattern. After the initial alkali-release stage, the Ca/Si ratio decreases rapidly while the *C_Ca_* in solution remains relatively high. A pronounced inflection point appears at approximately 61 mmol/L, where C/S drops sharply to around 0.72. As *C_Ca_* further decreases to approximately 43 mmol/L, the C/S ratio continues to decline significantly and approaches values close to 0.1. At lower C/S ratios, calcium concentration gradually approaches the background level of seawater.

During the early stage of calcium loss (Ca/Si > 2.62), the artificial seawater pH remains close to the buffering range of portlandite, and the reduction in C/S is mainly attributed to portlandite dissolution. Meanwhile, the coexistence of Mg^2+^, SO_4_^2−^, and Cl^−^ promotes the formation of secondary phases such as AFt, brucite, and Friedel’s salt. As the pH decreases and portlandite buffering capacity diminishes, further reduction in C/S is increasingly governed by C-S-H decalcification. During this stage, brucite continues to form, while Friedel’s salt begins to decompose and AFt transitions from formation to gradual dissolution.

A key feature of the seawater system is that a significant reduction in C/S occurs while the *C_Ca_* in solution remains relatively high, indicating a decoupling between solid-phase degradation and aqueous chemistry. As *C_Ca_* approaches the background level of seawater, the C/S ratio decreases to extremely low values, indicating a highly decalcified state of the solid. At this stage, AFt is nearly depleted, brucite stabilizes at a low level, and carbonate-type AFm phases remain as the dominant stable phases, marking the establishment of the final equilibrium state under low alkalinity conditions.

Overall, the comparison between NaCl solution and artificial seawater demonstrates that the solid–liquid equilibrium of WCP is strongly governed by the ionic composition of the surrounding environment. While the NaCl system follows a relatively conventional equilibrium-controlled decalcification sequence, the seawater system is dominated by coupled dissolution–precipitation reactions involving Mg^2+^ and SO_4_^2−^. This leads to a fundamentally different equilibrium behavior, characterized by rapid and extensive solid-phase decalcification occurring over a narrow range of calcium concentration, as well as a pronounced decoupling between solid degradation and aqueous chemistry. These results indicate that equilibrium relationships established in deionized water are not directly applicable to marine environments, highlighting the necessity of incorporating multi-ionic interactions and secondary phase formation into predictive models for the calcium leaching of Portland cement concrete exposed to seawater.

## 4. Conclusions

This study experimentally established the solid–liquid equilibrium relationship of calcium in hardened Portland cement paste exposed to NaCl solution and artificial seawater. Based on the obtained results, the following conclusions can be drawn:

(1) Both NaCl solution and artificial seawater significantly increase the equilibrium calcium concentration compared to deionized water. The maximum calcium concentration increases to 27.1 mmol/L in 0.53 M NaCl solution and 68.5 mmol/L in artificial seawater, indicating a strong enhancement effect of saline environments on calcium dissolution.

(2) In NaCl solution, the solid–liquid equilibrium follows a classical multi-stage decalcification process, including portlandite dissolution, gradual C-S-H decalcification, and subsequent decomposition of AFm and AFt phases. The overall evolution remains consistent with equilibrium relationships observed in pure water, but with elevated calcium solubility due to chloride complexation and ionic strength effects.

(3) In artificial seawater, the equilibrium behavior deviates significantly from that in NaCl solution. The presence of Mg^2+^ and SO_4_^2−^ induces strong coupled dissolution–precipitation reactions, including the formation of brucite, AFt, and M-S-H. These reactions accelerate calcium removal and result in rapid and extensive decalcification within a narrow calcium concentration range.

(4) The solid phase in seawater can undergo severe decalcification while the aqueous phase still maintains relatively high calcium concentrations, indicating a decoupling between solid degradation and solution chemistry. This behavior cannot be described by conventional equilibrium models derived from pure water systems.

(5) The experimentally established equilibrium relationships provide essential data for improving thermodynamic modeling and durability prediction of Portland cement concrete in marine environments. Future work should focus on integrating multi-ionic coupling effects into reactive transport models to better simulate long-term degradation processes.

## Figures and Tables

**Table 1:** Chemical composition of the cement.

Oxide	CaO	SiO_2_	Al_2_O_3_	Fe_2_O_3_	MgO	SO_3_	Na_2_O	K_2_O	Cl	Others
Content (%)	65.40	18.80	3.92	3.23	2.82	4.03	0.231	0.861	0.082	0.626

**Table 2:** The mass content in 1 L of artificial seawater.

Reagent	Deionized Water	NaCl	MgCl_2_·6H_2_O	MgSO_4_·7H_2_O	CaCl_2_·2H_2_O	KCl	Total Mass
Mass/g	983.500	27.378	5.084	6.896	1.470	0.745	1025.073

## Data Availability

The original contributions presented in this study are included in the article. Further inquiries can be directed to the corresponding author.
